# Ratio That Reveals Risk: Defining TG:HDL-C Ratio Cutoff for Insulin Resistance in Adolescents with Obesity

**DOI:** 10.3390/children13081098

**Published:** 2026-08-18

**Authors:** Ahmad Kamil Nur Zati Iwani, Muhammad Yazid Jalaludin, Abqariyah Yahya, Shazana Rifham Abdullah, Liyana Ahmad Zamri, Fazliana Mansor, Janet Yeow Hua Hong, Abdul Halim Mokhtar

**Affiliations:** 1 Endocrine and Metabolic Unit, Nutrition, Metabolic, and Cardiovascular Research Center, Institute for Medical Research, National Institute of Health, Ministry of Health Malaysia, Setia Alam 40170, Malaysia; shazana.a@moh.gov.my (S.R.A.); liyana.az@moh.gov.my (L.A.Z.); fazliana.m@moh.gov.my (F.M.); 2 Department of Paediatrics, Faculty of Medicine, University Malaya, Kuala Lumpur 50603, Malaysia; 3 Department of Social and Preventive Medicine, Faculty of Medicine, University Malaya, Kuala Lumpur 50603, Malaysia; abqariyah.yahya@um.edu.my; 4 Department of Paediatrics, Hospital Putrajaya, Ministry of Health Malaysia, Putrajaya 62250, Malaysia; ppjanet@hpj.gov.my; 5 Department of Sports Medicine, Faculty of Medicine, University Malaya, Kuala Lumpur 50603, Malaysia; drhalim@um.edu.my

**Keywords:** adolescents, obesity, insulin resistance, TG:HDL-C ratio, cutoff

## Abstract

**Highlights:**

**What are the main findings?**

• An optimal TG: HDL-C ratio cutoff of 0.89 was identified for detecting insulin resistance, with an AUC of 0.73, 62% sensitivity, and 75% specificity.
• The TG: HDL-C ratio demonstrated comparable discriminatory ability to the leptin:adiponectin ratio (AUC 0.73 vs. 0.68, *p* = 0.30), supporting its potential as a practical alternative based on routine lipid parameters.

**What are the implications of the main findings?**

• The TG:HDL-C ratio is a practical, lipid-based marker for identifying insulin resistance in children and adolescents with obesity.
• The proposed TG:HDL-C ratio cutoff offers a practical approach for early screening of insulin resistance in children and adolescents with obesity using routine lipid profiles.

**Abstract:**

**Background/Objectives**: Insulin resistance (IR) plays a central role in metabolic dysfunction and elevates the risk of chronic diseases such as type 2 diabetes mellitus in later life. Early identification of IR in adolescents is therefore important. This study aimed to determine the triglyceride-to-high-density lipoprotein cholesterol (TG:HDL-C) ratio cutoff for IR and assess its association with IR predictors among adolescents with obesity. **Methods**: A total of 215 blood samples from adolescents aged 13–16 years with obesity were analyzed for fasting blood glucose, lipids, insulin, leptin, and adiponectin. Obesity was defined using BMI z-scores, while IR was classified using the Homeostatic Model Assessment for Insulin Resistance (HOMA-IR ≥ 4.0). Receiver operating characteristic (ROC) analysis was performed to compare the discriminatory performance between the TG:HDL-C ratio and Leptin:Adiponectin ratio (LAR). Multiple logistic regression was performed to assess associations between the TG:HDL-C ratio and IR-related markers. **Results**: The area under the ROC curve for the TG:HDL-C ratio was comparable to LAR (0.73 vs. 0.68, *p* = 0.3). The optimal TG:HDL-C ratio cutoff identified using the maximum Youden index was 0.89, with 62% sensitivity and 75% specificity. A TG:HDL-C ratio ≥ 0.89 was associated with higher odds of elevated insulin (AOR 1.1, 95% CI 1.0–1.13), elevated HbA1c (AOR 2.7, 95% CI 1.1–6.6), abdominal obesity (AOR 3.6, 95% CI 1.9–6.7), and raised LAR (AOR 1.2, 95% CI 1.0–1.5). **Conclusions**: These findings suggest that a TG:HDL-C ratio cutoff of 0.89 may be a useful marker for identifying IR in adolescents with obesity.

## 1. Introduction

The prevalence of childhood obesity is increasing globally. Malaysia is similarly affected, reporting one of the highest rates of childhood obesity in Southeast Asia [1]. Children with obesity are at markedly higher risk of developing insulin resistance (IR), type 2 diabetes mellitus (T2DM), cardiovascular disease (CVD), metabolic syndrome (MetS), and metabolic (dysfunction)-associated fatty liver disease (MAFLD) [2,3]. Timely identification of IR in at-risk adolescents is crucial to prevent the growing epidemic of childhood obesity and its associated complications. Early detection enables prompt interventions aimed at improving insulin sensitivity, such as lifestyle modifications involving diet, physical activity, and behavioural changes. Recent evidence suggests that the triglyceride-to-high-density lipoprotein cholesterol (TG:HDL-C) ratio is a convenient and accessible marker for identifying IR at an early stage in children with obesity [4,5,6]. This lipid-derived index reflects key aspects of the metabolic dysregulation commonly associated with IR, such as elevated triglycerides and reduced HDL-C levels, which are both features of atherogenic dyslipidemia [7,8].

While research on using the TG:HDL-C ratio as IR surrogate in children is emerging, it primarily focuses on establishing its association with other IR surrogates like homeostasis model assessment for IR (HOMA-IR) and impaired glucose tolerance across various populations [4,9,10], as well as its association with obesity-associated comorbidities such as IR [11], MetS [12,13], non-alcoholic fatty liver disease currently known as MAFLD [14] and CVD risk factor [15,16]. Currently, data on TG:HDL-C ratio cutoff values for predicting IR in the pediatric population remain limited [17]. Ethnic-specific analyses have highlighted variability in optimal cutoff points [4,18,19,20], reflecting differences in lipid metabolism and IR susceptibility among populations. For example, adolescents of Asian descent may demonstrate different TG:HDL-C ratio dynamics compared to their Western counterparts due to differences in body fat distribution and insulin sensitivity [21,22,23]. Thus, population-based studies and regional data remain essential to establishing standardized thresholds.

Establishing an appropriate cutoff value for the TG:HDL-C ratio is essential to optimizing its clinical utility as a surrogate marker for IR in adolescents. This study aims to identify the TG:HDL-C ratio cutoff point for IR and to examine its association with IR-related predictors in adolescents with obesity. Additionally, we also compare the TG:HDL-C ratio discriminatory power to an established adiposity–IR marker (platinum standard:leptin:adiponectin ratio) [24,25]. To the best of our knowledge, this is the first pediatric study on the TG:HDL-C ratio to perform this comparison.

## 2. Materials and Methods

Study Design and Participants: Data for this study were obtained from the baseline data of adolescents with obesity enrolled in the My Body is Fit and Fabulous at School (MyBFF@school) programme. A detailed recruitment process for MyBFF@school programme has been described previously [26]. Written informed consent was obtained from parents or legal guardians, and written assent was obtained from participating children. The consent form for participation was distributed to all participants and signed. The study was conducted in accordance with the Declaration of Helsinki, and the protocol was approved by the Medical Research and Ethics Committee (MREC), Ministry of Health Malaysia (NMRR-18-2749-41841) on 18 December 2018. All procedures adhered to the approved protocol and applicable ethical guidelines. In summary, MyBFF@school was a six-month school-based intervention study, aimed at combating the increasing prevalence of childhood obesity among Malaysian schoolchildren. The programme was conducted between February and August 2016 across schools in the Federal Territory of Kuala Lumpur, Selangor, and Negeri Sembilan of Malaysia. This present study included 215 pubertal adolescents [Table S1] with obesity, aged 13 to 16 years old, who had complete data on anthropometric measurements, pubertal staging, and blood assayed for leptin and adiponectin [12]. To minimize variability associated with pubertal development, the analysis was restricted to participants aged 13–16 years, as the majority of Malaysian adolescents have entered puberty by this age range. The required sample size was estimated using a sensitivity and specificity sample size calculator, assuming an expected sensitivity of 90%, specificity of 85%, and an estimated prevalence of IR of 30% among children with obesity [27]. Based on these assumptions, the minimum required sample size was 116 participants. The final sample of 215 participants substantially exceeded this requirement, indicating that the study was adequately powered to evaluate the diagnostic performance of the TG:HDL-C ratio and to provide precise estimates of the area under the ROC curve (AUC), sensitivity, specificity, and the optimal cutoff value. Data required for this study was assessed on 15 January 2019. All data were fully anonymized prior to author access. This study was approved by the Medical Research and Ethics Committee (MREC), Ministry of Health Malaysia (NMRR-18-2749-41841).

Health and Physical Examination: Each adolescent was assessed by medical officers and pediatricians who took a clinical history, performed a health examination including the presence of acanthosis nigricans (AN) [28] and a pubertal stage assessment [29,30]. Standardized anthropometric procedures were used to assess height, body weight, body fat mass, and waist circumference. Participants were measured while barefoot and wearing light clothing. Height was determined using a calibrated stadiometer (Seca 217, Hamburg, Germany). Body weight and body fat mass were assessed with a pre-calibrated bioelectrical impedance analyzer (InBody 720, Seoul, Republic of Korea), and all measurements were recorded to the nearest 0.1 kg. Waist circumference was measured twice using a non-stretch measuring tape (Seca 201, Hamburg, Germany) placed directly on the skin at the midpoint between the 10th rib and the iliac crest after normal expiration. The mean of the two waist circumference measurements was used for subsequent analysis. Blood pressure readings were taken twice at five-minute intervals using a mercury sphygmomanometer (Accoson, Irvine, UK), with the adolescent seated and the arm supported at the heart level.

Blood Sample Collection and Biochemical Measurements: Venous blood samples were obtained after an overnight fast of at least 8 h by trained nurses and physicians. Immediately after collection, the specimens were placed in a cooler containing frozen ice packs and transported to the central laboratory at the Institute for Medical Research within 2 h. All samples were processed on the day of collection. Serum and plasma were separated into aliquots and stored at −80 °C until analysis. Biochemical analyses included leptin, adiponectin, glycated hemoglobin (HbA1c), fasting plasma glucose, insulin, triglycerides, total cholesterol, high-density lipoprotein cholesterol (HDL-C), and low-density lipoprotein cholesterol (LDL-C). Detailed analytical methods for these biochemical parameters have been described in a previous publication [12].

Operational Definitions of Study Variables: Obesity Status; Overweight and obesity were defined as BMI z-score above 1 and 2 standard deviations for age and sex according to the WHO BMI chart [31]. Pubertal Staging; Tanner staging was self-assessed by participants with the aid of standardized Tanner staging diagrams. For boys, genital development and pubic hair development were assessed, while for girls, breast development and pubic hair development were assessed. Stage 1 external genitalia development and breast development for boys and girls were classified as prepubertal, while stage 2 and above were defined as pubertal [29,30]. Insulin Resistance Index; IR status was defined based on the HOMA-IR, calculated by multiplying the value of fasting plasma insulin (U/mL) and fasting plasma glucose (mmol/L) and then dividing by 22.5 [32]. Insulin Resistance Status; The score of HOMA-IR ≥ 4.0 was classified as IR, while a score of less than 4.0 was considered as insulin-sensitive [33]. The cutoff point of 4.0 was chosen because (1) this threshold has been used in previous studies involving children and adolescents with obesity [33]; (2) the lower limit of our top quantile of HOMA-IR distribution values is 4.13; and (3) prospective study has shown that subjects at the top decile of HOMA-IR demonstrated substantially greater risks of developing prediabetes and T2DM in adulthood [34,35]. Statistical Analysis: The normality of continuous variables was assessed using the Kolmogorov–Smirnov test. Continuous variables were reported as means and standard deviations (SDs). The differences in means between two groups were determined using the independent-sample *t*-test, while the Mann–Whitney test was applied for not normally distributed data . Categorical variables were compared using the chi-square test. Statistical significance was set at 0.05. A receiver operating characteristic (ROC) curve analysis was performed to evaluate the diagnostic performance of the TG:HDL-C ratio for discriminating between adolescents with insulin-sensitive and insulin resistance (IR). The area under the ROC curve (AUC) was calculated, and the optimal TG:HDL-C ratio cutoff value was determined using the maximum Youden index. An AUC of 0.5 indicates no discriminatory ability, 0.7–<0.8 indicates acceptable discrimination, and 0.8–<0.9 indicates excellent discrimination [36]. The Youden index was calculated as J = sensitivity + specificity − 1, with the optimal cutoff defined as the point that maximized the combined sensitivity and specificity [37]. Based on the optimal cutoff value identified from the ROC analysis, logistic regression analyses were performed to identify the association between high TG:HDL-C ratio and IR-related predictor, adjusted for gender. All statistical analyses were performed using IBM SPSS Statistics for Windows, Version 22.0 (IBM Corp, Armonk, NY, USA) and Stata Statistical Software: Release 14 (Stata Corp LP, College Station, TX, USA).

## 3. Results

Table 1 shows the general and clinical characteristics of the adolescents by insulin sensitivity. This study included 215 pubertal adolescents, ranging from 13 to 16 years old. Of the 215 adolescents, 36% (n = 77) were found to have IR, as defined by HOMA-IR ≥ 4. There was no difference in the number of boys and girls between groups. The majority of the adolescents were Malay (77.2%), followed by Indian (11.2%), Chinese (7.4%), and other minority ethnicities (4.2%). Regarding obesity status, 27.3% of adolescents with IR were morbidly obese, compared to only 2.9% in insulin-sensitive adolescents. Adolescents with AN (46.4%) and MetS (13.0%) were more common in adolescents with IR than in insulin-sensitive adolescents. Additionally, the mean systolic blood pressure was significantly higher (117 ± 12 mmHg, *p* < 0.01) in adolescents with IR compared to insulin-sensitive adolescents (110 ± 12 mmHg, *p* < 0.01).

Table 2 describes the biochemical characteristics of the adolescents by insulin sensitivity. Adolescents with IR had significantly higher medians of the TG:HDL-C ratio and LAR compared to insulin-sensitive adolescents (1.0 vs. 0.7, *p* < 0.01) and (2.3 vs. 1.4, *p* < 0.01), respectively. Likewise, all other parameters were significantly higher in the adolescents with IR group, except for the mean total cholesterol.

**Figure 1:**
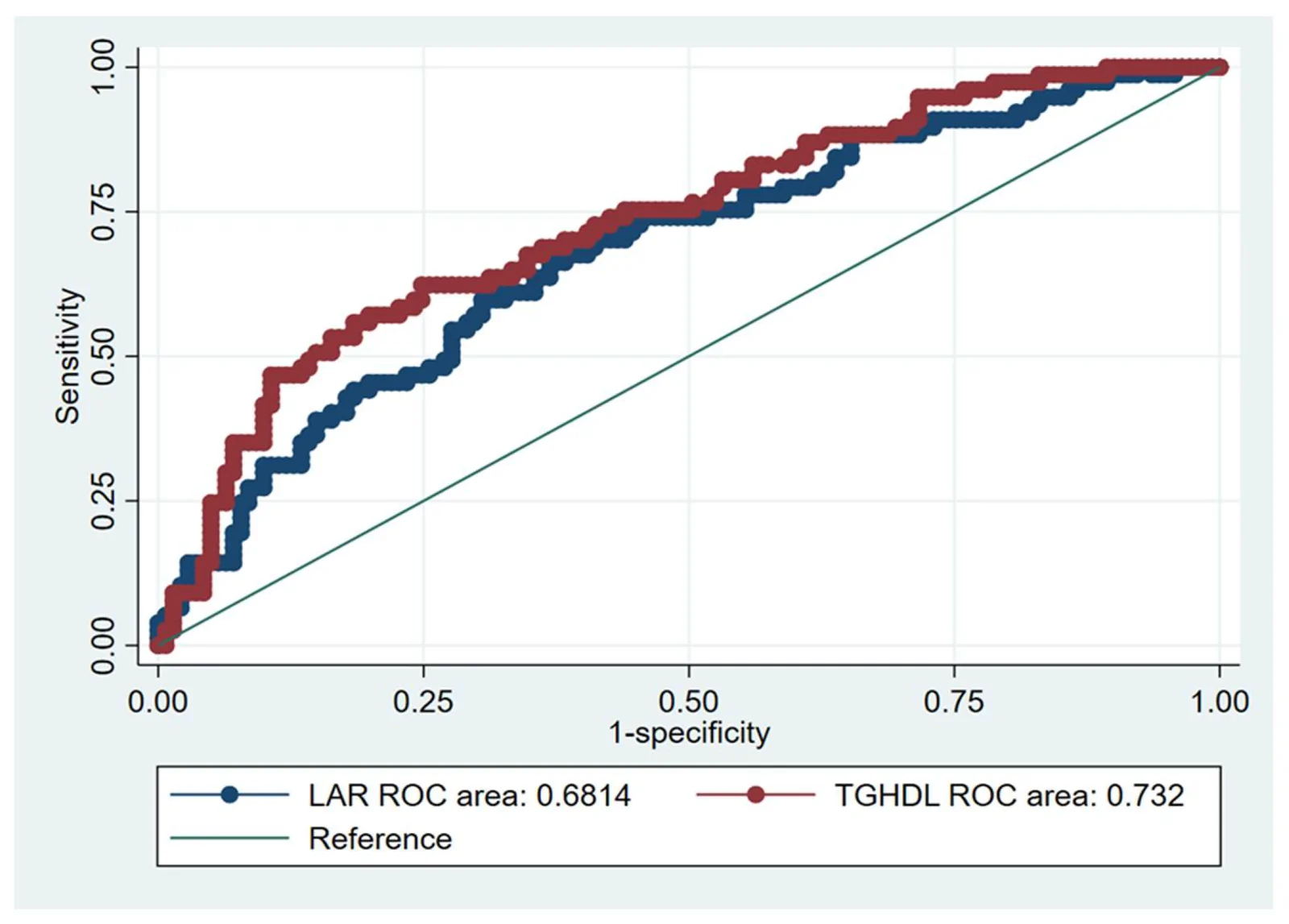
Comparison of receiver operating characteristic curves between TG:HDL-C ratio and Leptin:Adiponectin ratio to detect insulin resistance (HOMA-IR).

The AUC for the TG:HDL-C ratio to predict IR was comparable to LAR (0.73 vs. 0.68 *p* = 0.3) (Figure 1). The AUC of 0.73 (95% CI: 0.67–0.82) indicates good discriminatory power. The cutoff corresponding to the maximum Youden index was identified at 0.89. This cutoff value resulted in a sensitivity of 62% and a specificity of 75% (Table 3). When further exploring the performance of the TG: HDL-C ratio at different thresholds, we found that at lower cutoffs, sensitivity increased at the expense of specificity, while higher cutoffs led to improved specificity but reduced sensitivity.

Based on the cutoff points established by the ROC curve, an elevated TG:HDL-C ratio was categorized as ≥0.89, while an adequate ratio was defined as <0.89. Adolescents with an elevated TG:HDL-C ratio were 1.1 times more likely to have elevated fasting insulin (AOR 1.1, 95% CI 1.05, 1.13) and 2.7 times more likely to have elevated HbA1C (AOR 2.7, 95% CI 1.1, 6.6) (Table 4) compared to those with an adequate ratio. Additionally, adolescents with an elevated TG:HDL-C ratio were 1.2 times more likely to have a higher LAR compared to those with an adequate ratio (AOR 1.2, 95% CI 1.02, 1.5). Furthermore, those with an elevated TG:HDL-C ratio were 3.6 times more likely to have abdominal obesity compared to those with an adequate ratio (AOR 3.6, 95% CI 1.9, 6.7).

## 4. Discussion

The TG:HDL-C ratio has emerged as simpler and more accessible lipid-based indices for IR—particularly in children and adolescents with obesity [4,5,6]. However, the TG:HDL-C ratio clinical application in pediatric populations remains limited, and standardized cutoff values for identifying IR across different ethnic groups have yet to be established. The main finding of this study suggests that the TG:HDL-C ratio presents good discriminatory ability in identifying IR in adolescents. A cutoff value at 0.89 was found to be significantly associated with IR risk—independent of gender—highlighting its significant implications for public health initiatives and clinical applications. Utilizing the TG:HDL-C ratio to identify IR in children and adolescents offers a valuable opportunity to initiate early interventions aimed at improving metabolic health outcomes.

The AUC values observed in this study fall within the range reported in previous studies (0.7–0.8) involving children and adolescents with obesity [4,18]. It is important to note that this is the first study that compared the ability of the TG:HDL-C ratio and LAR in determining IR among adolescents with obesity. We found that the TG:HDL-C ratio has similar discriminatory ability in identifying IR to LAR (*p* = 0.32). LAR is regarded as a reliable and credible marker of obesity-related IR in the pediatric population. The potential utility of the LAR as a surrogate marker of IR in children has been explored in several studies [38,39]. Adipokines have been recommended as adipose tissue biomarkers by International Diabetes Federation in their “platinum standard” definition of MetS for research [40]. Leptin and adiponectin differ from almost all other adipocytokines in being secreted exclusively by adipocytes [41]. Given the association between obesity, adipose tissue dysfunction, and IR, the LAR has been investigated as a potential surrogate marker reflecting alterations in adipokine homeostasis and insulin sensitivity [42]. Despite their clinical relevance, due to their high cost, leptin and adiponectin tests are not routinely utilized in clinical settings. Therefore, the TG: HDL-C ratio can be used as an accessible, practical, and affordable diagnostic tool for various medical conditions, particularly those related to IR and metabolic disorders.

Ethnic variability in lipid metabolism underscores the importance of population-specific TG:HDL-C ratio cutoffs. Among Asian Indian adolescents, reference data demonstrate higher triglycerides and lower HDL-C compared to U.S. peers, despite similar BMI and LDL levels [43]. Similarly, South Asian youth in Western settings show a higher prevalence of dyslipidemia—with up to 54% exhibiting low HDL-C and 30% elevated TG levels—relative to European and Chinese peers [44]. Moreover, cardiometabolic risk often emerges at lower BMI and lipid thresholds in Southeast Asian populations, including early manifestations of T2DM and MetS [45,46]. Collectively, these findings indicate that Western-derived TG:HDL-C ratio thresholds may underestimate risk in Southeast Asian adolescents. Therefore, establishing locally validated cutoffs is critical to improving early risk stratification and ensure the clinical relevance of this surrogate marker in our population.

Limited studies have determined the cutoff for TG:HDL-C ratio in adolescents, particularly those with obesity in Southeast Asia where the population is ethnically diverse [4,11,12,15,47]. As previously reported, the TG:HDL-C ratio is greatly influenced by ethnicity [48,49], indicating the need for a race or ethnicity cutoff point of this index. Gianini et al. were the first to report a TG:HDL-C ratio cutoff of 0.99 (2.27 mg/dL), demonstrating 89% sensitivity and 45% specificity for identifying IR in White adolescents with obesity [18], while Zhang et al. proposed a lower thresholds (0.725) in Asian populations [4]. In our study, the TG:HDL-C ratio achieved a sensitivity of 62% and specificity of 75% for detecting IR. While the sensitivity falls below the conventionally accepted 70% threshold for standalone screening, it is noteworthy that comparable values have been reported in other cohorts. For example, Paulmichl et al., using the euglycemic–hyperinsulinemic clamp as the gold standard, demonstrated that the TG:HDL-C ratio achieved only modest discriminatory performance, with sensitivities in a similar range [20]. This suggests that the moderate sensitivity observed in our study is not unexpected, but rather reflects the inherent limitations of lipid-derived indices in fully capturing the complex physiology of IR. Importantly, the consistency of our findings with clamp-validated data reinforces the utility of the TG:HDL-C ratio as a practical surrogate marker, particularly in resource-limited settings where direct assessment of insulin sensitivity is not feasible.

In recent years, several lipid-related indices have been proposed as surrogate markers of IR, each with varying degrees of practicality and diagnostic performance. The TG:HDL-C ratio remains the most widely used due to its simplicity, low cost, and broad validation across pediatric populations; however, its sensitivity is modest and cutoff values often vary by ethnicity [49]. The triglyceride-glucose (TyG) index, which incorporates fasting glucose alongside triglycerides, has demonstrated superior predictive ability compared with the TG:HDL-C ratio in many cohorts, including children and adolescents [50], but requires additional biochemical assessment. More recently, the Single Point Insulin Sensitivity Estimator (SPISE) has gained attention as an insulin-independent marker, showing strong correlation with clamp-derived measures of insulin sensitivity and potential utility in obese adolescents [51], though pediatric cutoffs remain less established. Other indices, such as the METS-IR score, may offer higher accuracy [5] but are limited by their complexity and reduced feasibility in routine clinical settings. Collectively, these markers highlight a trade-off between practicality and precision; while the TG:HDL-C ratio offers broad applicability for screening, indices such as TyG and SPISE may provide added value in research and clinical scenarios where improved diagnostic accuracy is required.

In addition, our study reveals that adolescents with a TG:HDL-C ratio ≥ 0.89 are more likely to exhibit abdominal obesity, which is a key characteristic of MetS and IR. The observed association between abdominal obesity and IR is consistent with the well-established bidirectional pathophysiological relationship reported in the literature. This relationship is underpinned by interconnected mechanisms, including chronic inflammation and disrupted glucose metabolism [52]; however, the cross-sectional design of the present study does not permit inference regarding the directionality of this association. Adolescents with an elevated TG:HDL-C ratio also exhibited higher insulin levels—a hallmark of IR. Although the association was modest and of borderline significance, this finding reinforces the biological link between dyslipidemia and hyperinsulinemia [53] while highlighting the potential of the TG:HDL-C ratio as a practical early marker for metabolic risk in obese adolescents. Nevertheless, the borderline significance warrants cautious interpretation, and future studies with larger samples are needed to confirm this association. Elevated HbA1c levels further reinforce this finding, as HbA1c reflects long-term glucose control and is commonly elevated in individuals with impaired glucose metabolism or prediabetes [54]. Moreover, the increased LAR observed in this group suggests a dysregulation in the adipokine secretions. An elevated LAR indicates an imbalance between pro-inflammatory leptin and anti-inflammatory adiponectin, which contributes to the pathophysiology of IR [55]. Taken together, these findings support the utility of an elevated TG:HDL-C ratio as a practical and accessible marker for identifying adolescents at increased risk of IR and related metabolic disturbances, thereby facilitating earlier intervention to prevent the progression to T2DM and CVD. Although our current sample size and predominantly Malay cohort limited the ability to assess the predictive value of the TG:HDL-C ratio for IR across Malaysia’s major ethnic groups, these findings provide a foundation for future studies with larger, more diverse cohorts.

## 5. Conclusions

In conclusion, this study identified a TG:HDL-C ratio cutoff of 0.89 as good discriminator of IR in adolescent with obesity. A TG:HDL-C ratio of ≥0.89 was associated with other established predictors of IR, supporting its utility as a promising marker in our study population. Although participants were recruited from the community, which enhances the generalizability of the findings and reduces the risk of selection bias, this secondary analysis included only adolescents with complete anthropometric, pubertal-stage, and biochemical data. As a formal comparison between included and excluded participants was not performed, the potential for selection bias cannot be entirely excluded. Therefore, these findings should be validated in larger, independent, and more diverse cohorts. Furthermore, because this study employed a cross-sectional design, temporal relationships could not be established; therefore, the findings should be interpreted as evidence of association rather than causation. Thus, longitudinal studies are needed to establish the temporal directionality and to determine whether the TG:HDL-C ratio can reliably predict future IR and cardiometabolic outcomes in adolescents. Despite these limitations, the study provides valuable insights into the potential role of the TG:HDL-C ratio as a practical lipid-based indicator of IR among children and adolescents with obesity.

## Figures and Tables

**Table 1:** General and clinical characteristic of children with insulin-sensitive and insulin resistance.

Characteristic	Insulin-Sensitive	Insulin Resistance	*p*-Value	All (n,%)
n,%	138 (64)	77 (36)		215
Age (mean ± SD)	14.7 ± 1.1	14.7 ± 1.1	0.66 ^a^	
Gender, n (%)				
Boys	52 (59.1)	36 (40.9)	0.20 ^a^	88
Girl	86 (67.7)	41 (32.3)		127
Ethnicity				
Malay	107 (77.4)	59 (76.6)	0.08 ^b^	166 (77.2)
Chinese	12 (8.8)	4 (5.2)		16 (7.4)
Indian	11 (8.0)	13 (16.9)		24 (11.2)
Others	8 (5.8)	1 (1.3)		9 (4.2)
BMI z-score (n, %)				
Overweight (> 1SD)	74 (53.6)	19 (24.7)	<0.01 ^b^	93
Obese (>2SD)	60 (43.5)	37 (48.1)		97
Morbidly obese (>3SD)	4 (2.9)	21 (27.3)		25
Waist Circumference (cm), median (p25, p75)	81.5 (76.8, 87.0)	94.7 (84.0, 102.0)	<0.01 ^c^	84.2 (78.2, 94.7)
Abdominal Obesity				
WC < 90th centile	70 (50.7)	15 (19.5)	<0.01 ^b^	
WC ≥ 90th centile	68 (49.3)	62 (80.5)		
Acanthosis Nigricans, n (%)				
Present	67 (53.6)	58 (46.4)	<0.01 ^b^	90
Absent	71 (78.9)	19 (21.1)		125
Metabolic Syndrome, n (%)				
No Metabolic Syndrome	133 (96.4)	67 (87.0)	0.01 ^b^	
With Metabolic Syndrome	5 (3.6)	10 (13.0)		
Blood pressure (mmHg)				
Systolic Blood Pressure (mean ± SD) (mmHg)	110 ± 12	117 ± 12	<0.01 ^a^	113 ± 12
Diastolic Blood Pressure (mmHg), median (p25, p75)	70 (63,77)	7 (66,80)	0.13 ^a^	

^a^ Independent sample *t*-test ^b^ Pearson Chi Square ^c^ Mann–Whitney U-Test.

**Table 2:** Biochemical characteristic of children with insulin-sensitive and insulin resistance.

	Insulin-Sensitive	Insulin Resistance	*p* Value	All
Total cholesterol (mmol/L) (mean ± SD)	4.1 ± 0.7	4.3 ± 0.7	0.07 ^a^	4.2 ± 0.7
Fasting Plasma Glucose (mmol/L) (mean ± SD)	4.8 ± 0.4	5.0 ± 0.4	0.001 ^a^	4.8 ± 0.4
HbA1c (%)(mean ± SD)	5.1 ± 0.3	5.3 ± 0.3	0.002 ^a^	5.2 ± 0.3
Insulin (µU/mL) median, (p25, p75)	12.4 (8.9, 14.6)	25.7 (22.5, 30.1)	<0.01 ^b^	15 (11.1, 23.2)
Triglycerides (mmol/L) median (p25, p75)	0.7 (0.6, 0.9)	0.9 (0.8, 1.3)	<0.01 ^b^	0.8 (0.6, 1.1)
HDL-C (mmol/L) (mean ± SD)	1.1 ± 0.2	0.9 ± 0.1	0.002 ^a^	1.0 ± 0.2
LDL-C (mmol/L) (mean ± SD)	2.8 ± 0.6	3.1 ± 0.7	0.004 ^a^	2.9 ± 0.7
TG:HDL-C ratio, median (p25, p75)	0.7 (0.5, 0.9)	1.0 (0.7, 1.4)	<0.01 ^b^	0.8 (0.6, 1.1)
Adiponectin (µg/mL) median (p25, p75)	5.8 (4.6, 7.7)	5 (3.9, 7.0)	0.02 ^b^	5.6 (4.0, 7.1)
Leptin (ng/mL), median (p25, p75)	7.6 (4.4, 7.7)	12.3 (6.9,17.8)	<0.01 ^b^	9.1 (5.5, 15.1)
Leptin:Adiponectin ratio median (p25, p75)	1.4 (0.7, 2.4)	2.3 (1.3, 3.9)	<0.01 ^b^	1.7(0.9, 3.0)

^a^ Independent sample *t*-test ^b^ Mann–Whitney U-Test.

**Table 3:** ROC curve-derived sensitivities and specificities for different TG:HDL-C ratio cutoffs in identifying insulin resistance.

TG:HDL-C Ratio Cutoff	Sensitivity	Specificity	PPV	NPV
≥0.70	0.75	0.6	44.96	78.65
≥0.75	0.74	0.57	48.72	80.20
≥0.80	0.68	0.64	50.49	78.26
≥0.85	0.64	0.69	52.69	77.6
≥0.89	0.62	0.75	57.83	78.52
≥1.11	0.47	0.89	75.45	70.59
≥1.27	0.32	0.93	71.43	71.58
≥1.28	0.32	0.93	71.43	71.58
≥1.37	0.25	0.94	67.86	69.47
≥1.40	0.25	0.95	73.08	69.79

ROC, receiver operating characteristic; PPV, positive predictive value; NPV, negative predictive value.

**Table 4:** Association between elevated TG:HDL-C ratio (≥0.89) and insulin resistance related markers.

	Area Under Curve	95% Confidence Interval	*p*-Value
Leptin:Adiponectin Ratio	0.681	0.61–0.76	0.32
TG:HDL-C ratio	0.732	0.67–0.87	
	b	Adjusted OR (95%CI)	*p *-Value
	Fasting Insulin		
TG:HDL-C ratio < 0.89	REF		
TG:HDL-C ratio ≥ 0.89	0.09	1.1 (1.05, 1.13)	<0.01
	Fasting Glucose		
TG:HDL-C ratio < 0.89	REF		
TG:HDL-C ratio ≥ 0.89	−0.247	0.78 (0.37, 1.7)	0.52
	HbA1c		
TG:HDL-C ratio < 0.89	REF		
TG:HDL-C ratio ≥ 0.89	0.99	2.7 (1.1, 6.6)	0.03
	Abdominal Obesity		
TG:HDL-C ratio < 0.89	REF		
TG:HDL-C ratio ≥ 0.89	1.3	3.6 (1.9, 6.7)	<0.01
	Leptin: Adiponectin Ratio		
TG:HDL-C ratio < 0.89	REF		
TG:HDL-C ratio ≥ 0.89	0.21	1.2 (1.03, 1.5)	0.02
	Leptin		
TG:HDL-C ratio < 0.89	REF		
TG:HDL-C ratio ≥ 0.89	0.004	1.0 (0.9, 1.1)	0.81
	Adiponectin		
TG:HDL-C ratio < 0.89	REF		
TG:HDL-C ratio ≥ 0.89	−0.146	0.864 (0.76, 0.98)	0.03
	Acanthosis Nigricans		
TG:HDL-C ratio < 0.89	REF		
TG:HDL-C ratio ≥ 0.89	0.46	1.6 (0.9, 2.8)	0.12
	Diastolic Blood Pressure		
TG:HDL-C ratio < 0.89	REF		
TG:HDL-C ratio ≥ 0.89	0.01	1.0 (0.98, 1.0)	0.49
	Systolic Blood Pressure		
TG:HDL-C ratio < 0.89	REF		
TG:HDL-C ratio ≥ 0.89	0.02	1.0 (0.90, 1.1)	0.21

b, regression coefficients; OR, odds ratio; CI, confidence interval. Logistic regression models were adjusted for gender.

## Data Availability

The data presented in this study are available on request from the corresponding authors. The data are not publicly available due to privacy and legal reasons.

## Supplementary Materials

The following supporting information can be downloaded at: https://www.mdpi.com/article/10.3390/children13081098/s1, Table S1: Tanner Stage Distribution of Study Participants.

## Abbreviations

The following abbreviations are used in this manuscript:

IR	Insulin Resistance
TG:HDL-C	triglyceride-to-high-density lipoprotein cholesterol
HOMA-IR	Homeostatic Model Assessment for Insulin Resistance
ROC	Receiver operating characteristic
LAR	Leptin:Adiponectin
T2DM	type 2 diabetes mellitus
CVD	cardiovascular disease
MetS	metabolic syndrome
MAFLD	metabolic (dysfunction) associated fatty liver disease
AUC	area under the curve
Tyg	triglyceride-glucose
SPISE	Single Point Insulin Sensitivity Estimator
