# Supplementary material for: Ratio That Reveals Risk: Defining TG:HDL-C Ratio Cutoff for Insulin Resistance in Adolescents with Obesity
Source: Children (Basel). 2026 Aug 18;13(8):1098. doi: 10.3390/children13081098 (PMC13511121; doi:10.3390/children13081098)
Supplement: Supplementary file 1 [file children-13-01098-s001.zip › children-4367548-supplementary.pdf]

Supplementary Table S1: Tanner Stage Distribution of Study Participants

Tanner Staging	Frequency (n, %)
II	11 (5.1)
III	47 (21.9)
IV	117 (54.4)
V	40 (18.6)
